# Clinical Experiences and Mechanism of Action with the Use of Oxytocin Injection at Parturition in Domestic Animals: Effect on the Myometrium and Fetuses

**DOI:** 10.3390/ani13040768

**Published:** 2023-02-20

**Authors:** Míriam Marcet-Rius, Cécile Bienboire-Frosini, Karina Lezama-García, Adriana Domínguez-Oliva, Adriana Olmos-Hernández, Patricia Mora-Medina, Ismael Hernández-Ávalos, Alejandro Casas-Alvarado, Angelo Gazzano

**Affiliations:** 1 Animal Behaviour and Welfare Department, Research Institute in Semiochemistry and Applied Ethology (IRSEA), 84400 Apt, France; 2 Department of Molecular Biology and Chemical Communication, Research Institute in Semiochemistry and Applied Ethology (IRSEA), 84400 Apt, France; 3 Neurophysiology, Behavior and Animal Welfare Assessment, DPAA, Universidad Autónoma Metropolitana, Xochimilco Campus, Mexico City 04960, Mexico; 4 Division of Biotechnology—Bioterio and Experimental Surgery, Instituto Nacional de Rehabilitación-Luis Guillermo Ibarra Ibarra (INR-LGII), Tlalpan, Mexico City 14389, Mexico; 5 Facultad de Estudios Superiores Cuautitlán, Universidad Nacional Autónoma de Mexico (UNAM), Cuautitlán 54714, Mexico; 6 Department of Veterinary Sciences, University of Pisa, 56124 Pisa, Italy

**Keywords:** oxytocin, domestic animals, parturition, meconium staining, fetal asphyxia

## Abstract

**Simple Summary:**

Oxytocin is a hormone that plays an important role in parturition. For this reason, in animal production farms, the exogenous application of this substance is regularly used to help in reducing calving times. However, this hormone can indirectly reduce vitality in the newborn by a direct effect on the intensity of uterine contractions, inducing fetal hypoxia and affecting neonate mortality and thereby a significant economic loss when it is administered in high doses or at times when it is not required. For this reason, the aim is to review the proper administration of oxytocin in domestic animals.

**Abstract:**

Oxytocin is a key hormone for parturition and maternal traits in animals. During the peripartum period, the levels of endogenous oxytocin dictate physiological events such as myometrial contractions, prostaglandin production with the subsequent increase in oxytocin receptors, and the promotion of lactation when administered immediately after birth. While this hormone has some benefits regarding these aspects, the exogenous administration of oxytocin has been shown to have detrimental effects on the fetus, such as asphyxia, meconium staining, ruptured umbilical cords, and more dystocia cases in females. This review aims to analyze the main effects of oxytocin on myometrial activity during parturition, and its potential favorable and negative administration effects reflected in the fetus health of domestic animals. In conclusion, it is convenient to know oxytocin’s different effects as well as the adequate doses and the proper moment to administrate it, as it can reduce labor duration, but it can also increase dystocia.

## 1. Introduction

In all mammal species, birth is characterized by an increase of plasma estradiol, prolactin, and cortisol levels, and by the activation of the oxytocinergic system at the time of delivery [1]. Oxytocin (OXT) is a neuropeptide that presents a dual action neurohormone and neurotransmitter with a chemical structure composed of nine amino acids [2,3,4,5,6]. For this reason, the use of endogenous OXT has become popular in the veterinary field for reproductive management in domestic animals [7].

The synthesis of this hormone has several physiological effects on the central nervous system (CNS). According to Young and Zingg [8], OXT is a behavioral modulator and the abundant presence of oxytogenic neurons in the brain is responsible for the modulation of behavior in both sexes. Additionally, it is pointed out that OXT may be responsible for the stimulation of affiliative relationships in mammals and also in the the generation of bonds of the dam–calf during imprinting [9,10,11]. During parturition, due to the distribution of OXT receptors, oxytocin also participates in lactation and the development and strengthening of the maternal behavior [10,12,13,14]. Notably, OXT induces milk ejection and is recognized for its participation in the induction of synchronous and sustained uterine contractions during parturition. For this reason, it is considered the most important hormone during this process [15,16,17,18]. Of note, the word “oxytocin” came from Greek words meaning “quick birth” after Dale discovered its uterine-contracting properties in 1906 [19].

On the other hand, it is important to mention that OXT induces contractions in the myometrium in late pregnancy and thereby causes a temporary decrease in blood flow and transient episodes of fetal hypoxia [3]. In addition to this, the number of OXT receptors increases as gestation progresses [20] and it seems important to know that G protein-coupled receptors, such as the oxytocin receptor, could experience desensitization after repeated or prolonged stimulation [21]. More precisely, Robinson et al. [22], demonstrated in vitro that OXT-induced desensitization of myocytes to OXT stimulation occurs within 4.2 h and that this mechanism of desensitization involved OXT receptors.

OXT is present in different systems as it is synthesized in the ovaries, nervous system, testicles, placenta, and even in cardiac tissue [3,4,5,6]. OXT is primarily synthesized in magnocellular neurons of the paraventricular and supraoptic nuclei of the hypothalamus. It is stored in vesicles in the posterior pituitary for its further release into peripheral circulation after neurologic stimulation [23,24,25]. It is also produced by smaller parvocellular neurons of the paraventricular nucleus of the hypothalamus, which directly projects to many regions of the brain, notably the limbic system, with structures such as the amygdala, the hippocampus, the nucleus accumbens, and the medial preoptic area (MPOA) [26].

In dogs, OXT promotes maternal interest in puppies by reducing anxiety and stimulating maternal care [13,27]. Intracerebroventricular infusions of OXT stimulate maternal behavior in sheep [28]. In sows, it has been shown by Gilbert et al. [29] that peripheral OXT is not involved in nest-building behavior because the pre-partum rise in OXT secretion has been linked to the end of sow nest-building behavior, which occurs 1–6 h before birth [30].

It has been seen that in swine production farms, sometimes sows experience prolonged farrowing, postweaning anestrus, and estrus return, which can have a significant impact on the economic profits of the company [31]. Some hormones play an important role in the labor process during farrowing [32] and if the time of farrowing could be reduced by administering them, it would be important for piglet survival, as a delay in farrowing can increase stillbirth numbers [33,34]. In this sense, serum estradiol, as well as the concentration of OXT, prolactin, progesterone, and prostaglandins have been used [3,4,5,6,15,16,17,35,36].

For this reason, the administration of exogenous OXT is one of the most important resources regarding the management of animals during farrowing, and some demographic data mention that 83.1% of pig farms use oxytocin as the first resource in the management of farrowing [37]. This is because its application has been observed to significantly reduce labor time, between 12 and 20 min in most species, and it begins to take effect within the first 10 min after its administration [2,38,39]. This can be recognized as an immediate benefit in relation to costs and care time, because an increase in synchronous contractions facilitates the expulsion of the newborn and even the expulsion of fetal membranes [40].

The benefits of OXT administration could have led to its indiscriminate use due to little control and the apparent absence of adverse effects of this neuropeptide [41,42,43]. However, due to the greater knowledge of the participation of OXT in other physiological systems, notably in cardiovascular regulation [44], it could make evident the adverse effects that the exogenous administration of this hormone could bring with it [3,16]. In some studies, it has been seen that the increase in uterine contractions caused by exogenous use of OXT can reduce blood flow at the level of the fetal umbilical artery [45]. This can cause hypoxia, which can be associated with states of respiratory stress and lead to suffocation [46], and, due to these events, the fetal vitality in newborns could be decreased [47,48,49,50,51]. In dogs, for example, fetal asphyxia represents 60% of losses of puppies [52]; in swine production, there are reports that have shown that mortality caused by asphyxia is 5–10% when this hormone has been administrated [53], while in dairy cattle there is an incidence of asphyxia between 0.67–9.2% derived from the use of OXT [54].

Therefore, these undesirable effects should be considered prior to OXT administration to reduce the potential dystocia and intrapartum mortality that this would generate. For this reason, the present review aims to analyze the mechanism of action of OXT, its main effects on myometrial activity during parturition, and the potential favorable and negative effects on the fetus of domestic animals.

## 2. Oxytocin: Pharmacology and Clinical Application at Parturition in Domestic Animals

In small ruminants, the half-life of OXT has been found to range from 1.3 (sheep) to 22 min (goats) [55,56]. In horses, it has been reported that the administration of 25 IU of intravenous OXT allows a half-life of 5.89 min, with a clearance rate of 7.78 min [57]. A comparative study of the pharmacokinetics of OXT suggests that the parameters of bioavailability, clearance time, and half-life are similar in most domestic mammals, which could possibly be taken as a reference [58]. Due to these reported half-life data, it is suggested that OXT in rabbits and goats has extensive penetration into different tissues [59,60]. It is worth mentioning that a short half-life in some animals leads to questioning if labor can be induced with a single administration of OXT (Table 1) [61,62].

The distribution of OXT and its clinical effect may be related to the route of administration, since it is mentioned that the preferred routes are intravenous (IV) and intramuscular (IM) [69,70,71]. In a study carried out by Mota-Rojas et al. [67] in 50 sows in which they compared the effect of OXT administered by three different routes: IM, IV, and intravaginal, they observed that in the animals that received OXT intravaginally and intravenously, there was an increase in the number of stillbirths and intrapartum broken cords. However, in the IM administration, umbilical cord rupture could be due to increased uterine contractions. In another study carried out by the same authors, they confirmed that IM administration of OXT significantly increased the intensity of uterine contractions, decreasing their duration and farrowing by up to 40 min, but with adverse effects for the fetus (e.g., meconium-stained piglets when OXT is administered at early phases of farrowing) [48]. This could make evident that the IM and IV routes of administration allow OXT to reach its highest plasmatic concentration and exert its action in the target organ, in this case, the uterus.

In the case of cattle, Wagner et al. [64] studied five bovines (*Bos taurus*) that received a dose of 0.66 IU/kg of OXT intranasally. They found that the concentration of OXT in the blood increased by 63.3 pg/mL at 3.5 min and that it maintained a half-life of 12.1 min. This indicates that the intranasal route could be an alternative for the use of OXT. However, while several behavioral effects have been shown with this route [72,73], as far as we know, to date, there are no reports indicating that it may have any effect on uterine activity.

According to Hill et al. [66], the most used doses of OXT in sows are 5 IU, 10 IU, 20 IU, 25 IU, 30 IU, and 40 IU administered by one of the three common routes of administration, IM, IV and intravulvar. A study was carried out on 200 sows in which they were grouped into four groups according to the dose of OXT administered (1 UI/6 Kg being the high dose, 3.1 UI/9 Kg being the medium dose, 1 UI/12 Kg being the low dose and control group). They observed that there was an increase in the number and intensity of contractions during farrowing in animals treated with the high and medium doses compared to the low dose, but they had greater deleterious effects on the fetuses [74].

In horses, the use of OXT is reported in doses of 3.5 IU up to 75 IU IM for the induction of uterine contractions. Villani and Romano [75], evaluated the effect of three low doses (3.5 IU) of IM OXT in 350 standardbred mares, pregnant at term. They observed that the administration of OXT caused a foaling stimulation effect on the length of gestation: 51.3% of the animals responded to the first dose of OXT, 14.2% to the second dose, and 3.4% to the third dose. This means that the last dose allowed moderate efficacy for labor induction. Opposite results were obtained in another study on 16 mares in which they compared the effect of two doses of oxytocin (1.75 IU and 75 IU) for labor induction. The authors observed that animals receiving the highest dose of OXT had a shorter interval from oxytocin administration to delivery of the fetus compared to animals receiving the higher dose, but resulting in weak foals [76].

Consequently, OXT tends to have a short plasma half-life so that a single dose may not achieve a correct stimulation of myometrial contractions. However, this effect may depend on the route of administration, the IM and IV routes being the most used. The dose may also be responsible for the intensity of the effects at the uterine level and, as studied in human medicine, the mode of administration could also have influence, but it is not well studied in animals.

## 3. OXT: Its Mechanism of Action and Receptor Signaling in the Myometrium in Domestic Animals

**Figure 1:**
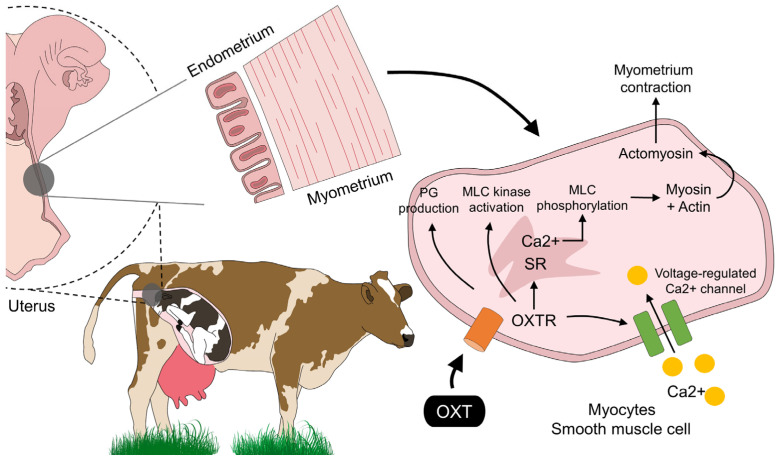
Oxytocin action on the smooth muscle cells in the myometrium. Endogenous and exogenous OXT promotes myometrium contraction by activation of the OXTR and its action on the voltage-regulated Ca^2+^ channels that facilitate Ca influx to the cell. In the SR, the interaction of Ca^2+^ participates in several events that result in muscle contraction, such as PG, production, MLC kinase activation, MLC phosphorylation, and the formation of actomyosin to maintain uterine contractions during calving: myosin light chain; MLC: myosin light-chain; OXT: oxytocin; OXTR: oxytocin receptors; PG: prostaglandin; SR: sarcoplasmic reticulum.

The mechanism of action of exogenous OXT in the myometrium does not differ from its endogenous ligand, promoting uterine contractions due to the arrangement of receptors in this tissue (Figure 1) [77]. In this regard, Taverne et al. [78] evaluated the functionality of uterine OXT receptors in cows around calving, using an intra-arterial dose of 800, 1600, and 3200 mU, making a continuous evaluation of the uterine electromyographic activity. The authors reported that OXT significantly increased myometrial activity and the magnitude of contractions. The production of uterine contractions in domestic animals is induced by the increase in intracellular Ca^2+^ in the myofibrils of the smooth muscle of the myometrium [60]. A close relationship between OXT receptor occupancy and Ca^2+^ entry through voltage-gated or coupled channels has been found, as suggested by in vitro studies on rat myometrial cells [3,79,80]. Furthermore, Riemer and Heymann [81] argue that, along with OXT and prostaglandins, connexin 43 (CX43) also participates in inducing myometrial contractility. Thus, it leads to understanding a possible stimulation of contractions through the activation of other contractile proteins such as CX43, due to the expression of genes encoding for proteins associated with a uterine contraction that cause an increase in said substance, which induces an ionic coupling to allow increased intracellular Ca^2+^ flow into myofibrils and coordinated contractions [82,83]. Immunohistochemical studies performed on neurons of the preoptic nucleus in lactating rats revealed that OXT neurons were positive for CX36, another protein, thus suggesting the involvement of this hormone with OXT [84].

The action of OXT can not only be observed at the uterine level. In fact, due to the universal distribution of its receptor, other physiological effects can be observed [16]. One of the most notorious would be the cardiovascular effect of OXT. Peterson [85] mentions that this neuropeptide induces a paradoxical effect at the vascular level because of its systemic vasodilator effect. In an in vivo study in nephrectomized Wistar rats, it was found that OXT attenuates the serum levels of urea, creatinine, and lactate dehydrogenase, which could be considered a protective effect [86]. However, at the uterine level, it can induce vasoconstriction [49]. Interestingly, given the abundant presence of OXT receptors in neurons, it has been suggested that it may be related to the mechanisms of nociception in females [12,87]. Biurrun Manresa et al. [88] suggest that this is possibly due to oxytocinergic neuron projections at the spinal cord that generate inhibitory modulation of painful stimuli. This makes it evident that OXT shows relevant physiological participation in different systems to consider that this hormone has a limited effect on the myometrium.

In summary, the mechanism of action of exogenous OXT allows an increase in the permeability of Ca^2+^ in the uterine myofibrils, which would stimulate contractions [89]. Despite its obvious mechanism of action, it has been argued that the efficacy of OXT may be affected by conditions such as a lack or decreased number of receptors in the myometrium [60]. In a systematic review, it was reported that the use of OXT reduces up to 3 to 5 min the farrowing interval time in piglets and up to 40 min the labor time of sows in the first stage of farrowing [66]. However, previous reports mention that OXT is not effective at term due to the absence of OXT receptors that develop after a change in the estrogen:progesterone ratio minutes before delivery [90].

Therefore, exogenous OXT promotes uterine contractions by occupying specific receptors and increasing intracellular ion flow in the myometrium. This mechanism of action is primarily responsible for the effect on the uterus. However, due to the dependence on the presence of receptors, the effect of exogenous OXT could be limited in the absence of said receptors. Another aspect relating to the OXT receptors could be of importance: their DNA sequences can be subject to variation, i.e., Single Nucleotide Polymorphisms, that can lead to OXT receptors variants, which in turn, may influence myometrial OXT response in the setting of parturition. For instance, an association between an OXT receptor variant and the development of postpartum hemorrhage in humans has been recently shown [91].

## 4. Favorable Effects of the Use of Oxytocin during Parturition and Recommendations for Its Use in Veterinary Obstetrics

Exogenous and endogenous OXT also has positive effects both in the dam and the fetus. For example, through placentophagy and consumption of amniotic fluids during parturition, the dam can obtain OXT, and some analgesic effects have also been reported [92]. In bitches, the administration of OXT during partum intervenes in the frequency of myometrial contractions, increasing them. It is usually given when uterine contractions are less than expected and the fetal heart rate is normal. However, the administration of low IV doses is recommended to prevent tetanic effects in the uterus, putting oxygenation of the fetus at risk [93,94]. According to Davidson [95], medical management of dystocia with irregular contractions or primary uterine inertia in bitches could include the administration of 10% calcium gluconate (0.465 mEq/4.5 kg SC) followed by OXT (0.5–1 U.S.P. units/bitch SC).

In the case of intensive pig production farms, the problems of sows at farrowing are resolved indistinctly through the use of oxytocics. However, it is important to point out that the application of exogenous OXT is not recommended in the following cases [96]: when there are regular contractions, when the cervix is not yet fully dilated, when there is a disproportion between the size of the fetus and the pelvis bone, when there is a bad presentation, and when there is any bleeding, vaginal prolapse, or hypocalcemia. In these cases, it can have adverse results if it is used neglectfully and indiscriminately [92]. In a study carried out by González-Lozano et al. [97] in sixty hybrid Yorkshire–Landrace sows (30 with eutocic farrowing and 30 with dystocia farrowing), in which was administrated exogenous OXT, at the dose of 0.083 UI/kg, to 15 eutocic and 15 dystocic sows, intramuscularly after the delivery of the 5th piglet. The results showed that OXT decreases the number of intrapartum deaths by 50%, and the highest viability was observed in the group of eutocic sows treated with OXT. Likewise, Paccamonti [98] points out that the application of OXT in mares for inducing foaling, with a single low dose of 10 IU, IV for a 550 kg mare is preferred because it is sufficient to initiate the cascade of events leading to foaling and provides a more natural course of events than higher doses or repeated administration. Therefore, it can be concluded that both the moment of application, as well as the dose, route, manner, and type of delivery influence whether there are benefits or adverse factors with the exogenous application of this hormone.

On the other hand, it is necessary to point out the importance of uterine contractions due to OXT, since, thanks to them, the exit of the lochia is favored, causing a more rapid uterine involution [99]. In the same way, this hormone contributes to stimulating the descent of milk into the mammary glands together with the stimulation that the offspring make when suckling [100], and also with the establishment of bonding between the dam and newborn [9,13,101,102,103].

Another important effect of OXT is that it can intervene in helping to establish maternal behaviors and decreasing the incidence of maternal cannibalism. This was confirmed in a study carried out by Kockaya et al. [104] in 30 adult Kangal bitches, 15 with the presentation of cannibalism and 15 that did not present it, in which the levels of OXT in serum were measured and it was found that in bitches with presentation of cannibalism, OXT levels were lower (3.58 ± 0.43 ng/mL) compared to bitches that did not present it (9.68 ± 1.58 ng/mL). In a study conducted by Feldman et al. [105] in humans, it has been shown that plasma and salivary OXT levels are associated with dam–infant bonding. Contrasting to these data, there was a study carried out by Ogi et al. [106] on 25 lactating Labrador Retriever dogs, in which salivary concentration of OXT was unrelated to the amount of maternal care, but had a weak negative correlation with sniffing/poking behavior. Therefore, salivary OXT concentration cannot be considered a strong predictive biomarker of the quantity of maternal care in dogs. Therefore, this study suggests that if what is sought is to improve maternal care in bitches, the exogenous application of OXT is probably not effective. Furthermore, the OXT assay procedures can differ and be themselves a source of discrepancies between the studies. Indeed, the assessment and interpretation of urine and saliva measurements often provide inconsistent findings. To a lesser extent, it can be also true for plasma measurements, as the methodological procedures can deeply impact the outcome of OXT concentrations [107,108].

Thus, it can be deduced that the application of OXT could have beneficial effects, if there are no situations such as those mentioned above in which its application is contraindicated.

## 5. Effects of the Exogenous Application of Oxytocin on the Fetus and the Umbilical Cord

**Figure 2:**
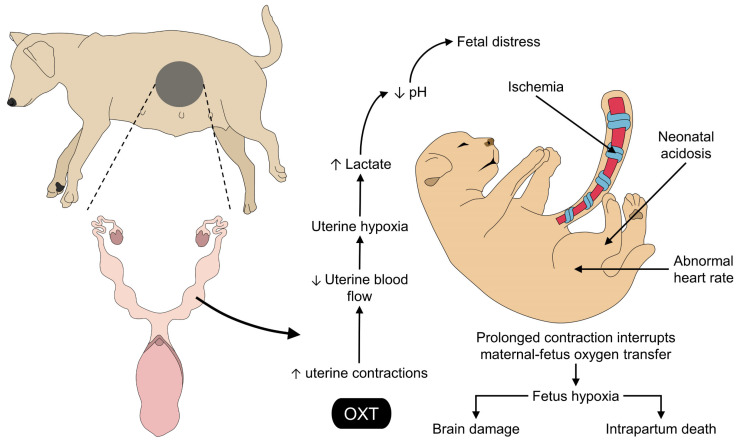
Effect of oxytocin administration on the umbilical cord and fetus health. Although OXT is commonly used during the onset of parturition, adverse effects directly on fetal health have been reported. After the hyperstimulation of the myometrium, prolonged contractions reduce uterine blood flow, resulting in a hypoxia state and accumulation of lactate. These events cause fetal distress with the main consequences including neonatal hypoxia, acidosis, abnormal heart rate, umbilical cord ischemia, and possible brain damage or intrapartum death when administering OXT. OXT: oxytocin.

The induction of myometrial contractions is the main reason behind the use of OXT during the parturition process [109]. Although the expulsion time is reduced, an increase in myometrial contractions due to the administration of OXT is a factor associated with decreased fetal oxygen saturation which can affect the viability and vitality of the fetus [110]. Moreover, its use can have an adverse effect on the fetus due to contractions, resulting in compromised blood flow (Figure 2) [111]. According to Mota-Rojas et al. [96], OXT given to sows at the onset of fetal expulsion significantly increases the rate of fetal distress, anoxia, and intrapartum death in piglets. The rupture of the umbilical cord, as well as the presence of meconium staining, could be other consequences of the excessive or misused exogenous application of OXT [65,112,113]. This effect on the fetus may be related to the type of placentation in different mammalian species [114]. For example, horses, cattle, and pigs present an epitheliochorial-type placenta, and some immunohistochemical studies have shown the presence of OXT receptors, mainly in the endometrium; however, the presence of these receptors in the allantochorionic or cotyledonous region is low, which possibly interferes with its impact on fetal membrane rupture [115,116]. In the case of species thar present hemochorial placenta, such as humans or rats, it has been suggested that OXT may lead to development of oxidative stress effects, due to the interrelationship that exists between the fetal vascular endothelium and the dam. Additionally, uterine contractions could reduce flow due to the rupture of this relationship [117].

In Yorkshire x Landrace sows, Alonso-Spilsbury et al. [47] found that the administration of a single dose of OXT immediately after the birth of the first piglet resulted in several health outcomes for the fetus, although the expulsion interval was also reduced from an average of 27.76 ± 0.81 min to a minimum of 22.2 ± 1.80 min. The presentation of stillbirths was higher in the first and third delivery of sows receiving OXT (a range between five and ten stillbirths), and the grade of meconium staining was greater (between 0.27 and 0.30). Additionally, an increased frequency of ruptured umbilical cords was observed in the treated animals compared to the control group (0.42–0.47 vs. 0.07, respectively), and dystocia cases were more prevalent in the sows receiving OXT (*p* < 0.01) [47].

The association between OXT and dystocia is due to the effect that OXT has on the intensity and duration of myometrial contractions [96], reducing uterine blood flow and fetal oxygenation [118]. This has been reported in 300 dairy Holstein calves, resulting in animals with lower mean oxygen partial pressure (39.6 ± 9.3 mmHg) (15 mmHg lower than control animals) and higher sodium concentration (137.7 ± 2.1 mmol/L) than newborns from dams not treated with OXT [118]. Therefore, these effects could affect neonatal vitality and welfare [12].

The direct effect of OXT on the local bloodstream has been suggested as one of the main adverse effects of its exogenous administration. For example, Stenning et al. [49] evaluated the effect of said hormone as an inducer of uterine contractions on the umbilical arterial and venous flow of premature lambs. The authors found that, together with an increase in myometrial contractions and blood pressure (of 5 ± 0.8 mmHg), there was a decrease in arterial and venous umbilical blood flow (by 12.1 ± 2 mmHg and by 20 ± 2 mmHg, respectively). These results show that the increased uterine contraction can diminish the blood flow and result in low systemic oxygenation and low newborn vitality. Likewise, a systematic review by Muro et al. [119] was carried out to evaluate the effect of uterotonics (OXT and carbetocin) on the duration of farrowing, farrowing interval, farrowing assistance, and feasibility of farrowing in sows. These authors observed that OXT reduced the duration of labor by 18% but increased the need for delivery assistance by 137% (*p* < 0.01). These figures were related to the 30% increase in stillborn pigs. For this reason, it seems that OXT would be a useful tool under strict technical guidance. However, there would still exist a risk of some harmful effects on the calf and the dam. The integrity of the umbilical cord and the uterus are other structures that can be affected by OXT administration. For example, in 2099 newborn piglets, the administration of OXT at the onset of fetal expulsion resulted in a higher incidence of stillborns, a greater piglets with ruptured (from 0.55 to 0.73) and hemorrhagic umbilical cords (from 0.21 to 0.36), together with an increase in the severity of meconium staining (*p* < 0.001) [96]. Fetal asphyxia has also been observed in 180 sows treated with OXT, resulting in a high percentage of umbilical cord rupture (76.0%) and an absence of heart rate in 53.5% of newborns [53]. Contrarily, in a murine model of fetal asphyxia (exposition of immature rats to 9% oxygen and 20% CO_2_), OXT administered intranasally to the newborn resulted in a neuroprotective role, preventing hippocampal injury [120]. Fetal distress, reported as meconium-stained newborns, was reported in piglets, with 2.5 times the presentation in litters from dams IM-treated with OXT at high doses (0.17 IU/kg), with an opposite effect being observed when low doses of 0.083 IU/kg were administered, also decreasing piglet mortality [65]. A beneficial effect was also reported in foals, in whom a mare receiving low doses of OXT resulted in foaling induction without the birth of immature foals [121]. Therefore, not only OXT but also the dose needs to be considered when deciding to use it during parturition.

The species must be considered in order to evaluate the effect that OXT might have on both the dam and the offspring. However, repeated administration of OXT during parturition causes hyperstimulation, leading to uterine exhaustion and reduced blood flow to the umbilical cord [122], and even uterine rupture when other confounding factors are present, such as an insufficient amniotic fluid [123] and limited cervical dilatation [124]. OXT-induced rupture leads to fetal distress and, consequently, high mortality of neonates and the need for medical intervention [125]. Therefore, the increase in uterine contractions could be associated with an increase in local blood pressure, which would lead to reduced blood flow, fetal stress, and hypoxia, and all these factors could reduce vitality in the newborn, having a high impact on its mortality [121]. In a case of a Great Dane bitch treated with repetitive doses of OXT and manual intervention to help in the dystocia process, a uterine rupture was reported [126]. The indiscriminate use of OXT can cause umbilical cord morphological alterations such as edema, congestion, hemorrhage, and, in extreme cases, umbilical cord rupture. High doses of oxytocin increase fetal heart rate decelerations and the presentation of meconium-stained fetuses. These elements are indicators of fetal hypoxia and can be reflected in intrapartum deaths [64]. Additionally, administering OXT at the beginning of parturition in polytocous species can double the number of meconium-stained fetuses and stillbirths during the first hour after administration [46]. It is relevant to mention the role of progesterone, a hormone that decreases during parturition to promote uterine contractions (known as the removal of the “progesterone block”) and fetal expulsion [127]. However, when administered in combination with caffeine at the last week of gestation in sows, the number of live born piglets decreased (11.7 ± 1.03 vs. 14.5 ± 0.73 in the control group) and piglet survival was impaired during the first five days of life and at weaning [128].

In horses, it is recorded that the contractions induced by the administration of OXT help to induce labor or expulsion of the placenta, which avoids the possibility of infections [76,129]. However, Sgorbini et al. [130] evaluated the effect of OXT at a dose of 2.5 IU IV on the incidence of peripartum complications at the behavioral, physical, and blood levels in mares. These authors found that foals born to mares under OXT treatment took longer to stand up and suckle compared to those born with spontaneous partum. In addition to this, they found higher levels of pCO_2_ and lactate. This evidence reaffirms that even though these changes are not considered clinically relevant in the dam, they can affect the physical condition of the newborn. In fact, at a high dose (3.5 UI), they can induce the birth of premature foals, thereby altering their vitality [121]. In horses, it is suggested that the presence of complications could possibly be related to pharmacological aspects, either due to the dose used and/or the frequency of administration [131].

Hence, when using OXT in a parturient female, it is essential to consider the benefits but also the possible adverse effects on the newborn, since the use of the hormone can fasten the parturition process but might result in negative outcomes for the fetus.

## 6. Future Directions

Due to the multiple effects that OXT administration can have on the dam and the fetus, the studies aiming to evaluate this hormone need to focus on different aspects of animal reproduction. One example is the role that OXT might have on behavior in both sexes. It is known that endogenous OXT participates in, and is a key element for, maternal bonding [132]. It has even been pointed out that it can regulate affiliative behavior between congeners. Additionally, alterations in the oxytocinergic system can alter postpartum maternal performance and the administration of exogenous OXT in the early stage has been proposed as a potential treatment to restore the functionality of OXT [133]. In bovines, the administration of OXT can facilitate social interaction, especially during maternal behavior, in addition to generating milk letdown [134,135]. Likewise, neglect and OXT administration to prevent this abnormal maternal behavior is being studied in humans with imaging techniques such as functional magnetic resonance to determine the cerebral regions activated by OXT, such as the inferior frontal junction and putamen [136,137]. In cases of male animals, this could be a field of study when the dam rejects the offspring or shows aggression towards them [25]. The circuit mechanisms by which oxytocin modulates social behavior are receiving increasing attention. Nonetheless, the complexity of the subject requires an entire article that is simultaneously produced by the authors of the present review (11).

The administration route and the pharmacokinetics of OXT depending on this aspect need to be studied to determine if, for example, subcutaneous administration of OXT also increases myometrial contractions and the risk of fetal distress. Currently, intranasal OXT to reach the brain and cerebral structures is being researched in humans [138] and non-human primates [139], but its association with fetal consequences during parturition needs to be determined. As it has been studied in humans, the mode of administration (i.e., bolus or infusion, or a combination of both) and its influence on labor complications [140,141] remains as another field that could be researched in veterinary medicine. In humans, authors such as Grotegut et al. [142] found that infusion of oxytocin was associated with severe postpartum hemorrhage, and Sheehan et al. [143] determined that the combination of bolus + infusion of oxytocin reduced the presentation of major obstetric hemorrhage in women. For this reason, the administration of OXT in different modes and its effect on myometrial activity and obstetric outcome could be assessed.

The implementation of other monitoring techniques, such as tocodynamometry, to evaluate and promptly detect OXT action on the myometrium smooth muscle cells is an alternative to continuing using OXT during parturition in domestic animals and to detect any uterine physiological compromising. Tocodynamometry has been used in the last decade in diverse species to detect primary uterine inertia [33]. Therefore, its implementation could help to identify the presence or absence of uterine myometrial contractions and their relative strength and frequency [95,144], and, if necessary, to determine whether or not the exogenous application of OXT is adequate to promote the presence of contractions. In the same way, it is necessary to study the relationship between the toxicological aspects of OXT with the impact that this hormone can have on the fetus or offspring, since the evidence is scarce in this regard.

Finally, it is important to teach producers and stock people that excessive use of OXT in parturient females must be managed prudently. An alternative could be imparting small courses taught by veterinarians to farmers and people involved with livestock so they can be aware of the advantages and disadvantages of the use of this hormone.

## 7. Conclusions

There are various functions of OXT around parturition, among which we can mention the establishment of bonding between the dam and offspring; myometrium contractions that, in addition to helping in the expulsion of the fetus, also intervene in the expulsion of lochia and placental residues; establishment of maternal behavior, as well as intervention in lactation by stimulating the descent of milk from the mammary gland. This is why in animal production farms this hormone is used indiscriminately to re-duce parturition times, as well as to reduce the incidence of dystocia caused by primary uterine inertia. Before OXT application, it is important to consider the weight of the dam, parity (e.g., primiparous or multiparous), the evolution of parturition, and if the birth canal is free, among other aspects.

There must be adequate management of the dose of OXT supplied, since with an excessive dose, the contractions become powerful and frequent, triggering heart rate deaccelerations and umbilical cord damage, producing partial fetal asphyxia and with each successive contraction, the situation may worsen, causing meconium-stained fetuses and weak, hypoglycemic neonates, until it causes fetal death. For this reason, when this hormone is used, it is convenient to know its effects, because, although in general terms, oxytocics reduce the duration of labor, they have the disadvantage of increasing dystocia. OXT dose-finding studies, as well as studies about administration routes and procedures, could be performed in each species of interest to clarify what doses and procedures are required for adequate effects on uterine contraction without side effects on the fetus.

## Figures and Tables

**Table 1:** Pharmacokinetic parameters of oxytocin in different species.

Dose	Absorption Kinetics		Pharmacokinetics	Species	Reference
	Administration Route	Evaluated Parameters			
25 IU	IV	T_1/2_: 5.89 min., clearance rate of 11.67 L/min., and mean residence time of 7.78 min.	Maximum effective concentration of 0.45 ng/mL and a plasma concentration of 0.25 ng/mL	Mare	Steckler et al. [57]
10 IU	IM	Minimal plasma concentration at 20 min. of administration 4822 pg/h/mL.	Tmax = 1.13 ± 0.91 h, Cmax = 2662 ± 567 pg/mL, AUC = 4822 ± 728 pg/h/mL, T_1/2_ = 1.02 ± 0.33 h.	Rabbit	Zhu and Lal [63]
400 IU	Sublingual	Plasmatic concentration at 20 min. of administration 1234 pg/h/mL.	Tmax = 0.93 ± 0.64 h, Cmax = 1164 ± 1179 pg/mL, AUC = 1234 ± 1001 pg/h/mL, T_1/2_ = 0.90 ± 0.33 h.		
0.33–1.32 IU	Intranasal	T_1/2_ = 12.2 min., Tmax = 12.2 min., Mean plasma concentration = 18.9 pg/mL.	Cmax = 77.3 pg/mL, AUC = 1726 pg/mL/min., mean residence time = 20 min.	Cow	Wagner et al. [64]
0.083, 0.11 and 0.17 IU	IM	T_1/2 =_ 1–6 min. Bioavailability: 70–100%, Vss: 40 min.	Clearance rate 7.87 mL.	Sow	Mota-Rojas et al. [65], Hill [66]
40 IU 20 IU	IM IV Intravulvar	T_1/2 =_ 1.94 ± 0.21 min.	Vd = 0.46 ± 0.02 L/kg, T_1/2_ of elimination = 22.3 ± 0.3 min.	Sow	Mota-Rojas et al. [67], Ybarra Navarro [68]

In the case of non-domestic species, the reported values for baboons receiving 500 IU IV OXT were a mean plasma concentration of 10 pg/mL on 10 min., T_1/2_ = 1.1 ± 0.2 min, Vd = 80 mL/Kg, Vss = 200 mL/Kg, fraction of elimination = 34 ± 5%, and mean residence time = 7.7 ± 0.8 min. For rats receiving 200–1000 ng/Kg IV, T_1/2_ = 21.09 min, T_1/2_ elimination = 7.94 min, Vd = 0.13–0.80 L/Kg, Vss = 0.34–0.80 L/Kg, and AUC = 2219–80,087 h/ng/L. AUC: area under the plot of plasma concentration of a drug versus time after dosage; Cmax: peak plasma concentration; IU: international units; IM: intramuscular; IV: intravenous; T_1/2_: half-life; Tmax: time to reach maximum concentration; Vd: volume of distribution; Vss: steady state volume of distribution.

## Data Availability

Not applicable.
